# The complete mitochondrial genome of *Cyclocheilichthys enoplos* (Teleostei, Cyprinidae)

**DOI:** 10.1080/23802359.2018.1437825

**Published:** 2018-02-12

**Authors:** Dehuai Luo, Jinjin Wang, Zuogang Peng

**Affiliations:** Key Laboratory of Freshwater Fish Reproduction and Development (Ministry of Education), Southwest University School of Life Sciences, Chongqing, China

**Keywords:** Mitochondrial genome, *Cyclocheilichthys enoplos*, Cyprinidae

## Abstract

Here, we reported the complete mitochondrial genome of *Cyclocheilichthys enoplos*, which is widely distributed in Southeast Asia. The genome was 16,579 bp in length, containing 2 ribosomal RNA genes, 22 tRNA genes, a non-coding control region (D-loop), and 13 protein-coding genes. The overall base composition of *C. enoplos* was 33.1% for A, 24.5% for T, 14.9% for G, and 27.5% for C. Phylogenetic analysis showed that all *Cyclocheilichthys* species clustered together formed a monophyletic group. The complete mitogenome data of *C. enoplos* would provide a basis for further systematic studies of the genus *Cyclocheilichthys*.

The freshwater and potamodromous fish *Cyclocheilichthys enoplos* (Bleeker 1849) is a cyprinid species, which is widely distributed in Cambodia, Indonesia, Laos, Malaysia, Thailand, and Viet Nam (Froese and Pauly 2018). Currently, the genus *Cyclocheilichthys* consists of eight valid species (Froese and Pauly 2018), but the complete mitochondrial genome data were only available for two species.

Here, we determined the complete mitogenome sequence of *C. enoplos*. Samples were collected from Tonle Sap Lake (13.24N/103.83E) and preserved in the museum of the Key Laboratory of Freshwater Fish Reproduction and Development (Southwest University, China). The methods for assembling the sequence followed the procedures outlined in Wang et al. (2011). In order to explore the phylogenetic position of *C. enoplos*, we selected another 11 species for phylogenetic analysis using *Puntius semifasciolatus* as an outgroup (Yang et al. 2012). Sequences were aligned using ClustalW algorithm in MEGA7 (Kumar et al. 2016). RAxML version 8.2.9 (Stamatakis 2014) was used to construct Maximum-likelihood (ML) trees with the GTR + Γ + I model. Branch support for the resulting phylogeny was evaluated with 500 rapid bootstrapping replicates also implemented in RAxML.

The whole mitogenome of *C. enoplos* was a circular molecule with 16,579 nucleotides (GenBank accession number NC_023231), containing 2 ribosomal RNA genes, 22 tRNA genes, a non-coding control region (D-loop), and 13 protein-coding genes. Most of which were encoded on the heavy strand, except for *ND6* and eight tRNAs (*tRNA^Gln^*, *tRNA^Ala^*, *tRNA^Asn^*, *tRNA^Cys^*, *tRNA^Tyr^*, *tRNA^Ser^*, *tRNA^Glu^*, and *tRNA^Pro^*). The contents of A, T, G, and C are 33.1%, 24.5%, 14.9%, and 27.5%, respectively. A + T content (57.6%) was higher than G + C content (42.4%). All of the 13 protein-coding genes show the regular initiation codon ATG with the sole exception of *COI* (started with GTG). Furthermore, the stop codons for 5 of the 13 protein-coding genes are TAA (*ND1*, *ATP6*, *ND4L*, *ND5*, and *cyt b*) and two of them are TAG (*ATP8* and *ND6*), whereas other five genes possess incomplete stop codons TA– or T–– (*ND2*, *ND3*, *ND4*, *COII*, and *COIII*). Specially, *COI* is end with AGG.

The results from the phylogenetic analysis strongly support that the three *Cyclocheilichthys* species forms a monophyletic group, and *C. janthochir* has a close relationship with *C. heteronema*. However, the genus *Barbonymus* is not a monophyletic group because the species *B. gonionotus* is clustered with *Mystacoleucus marginatus*, rather than cluster with other *Barbonymus* species (Figure 1).

**Figure 1.**
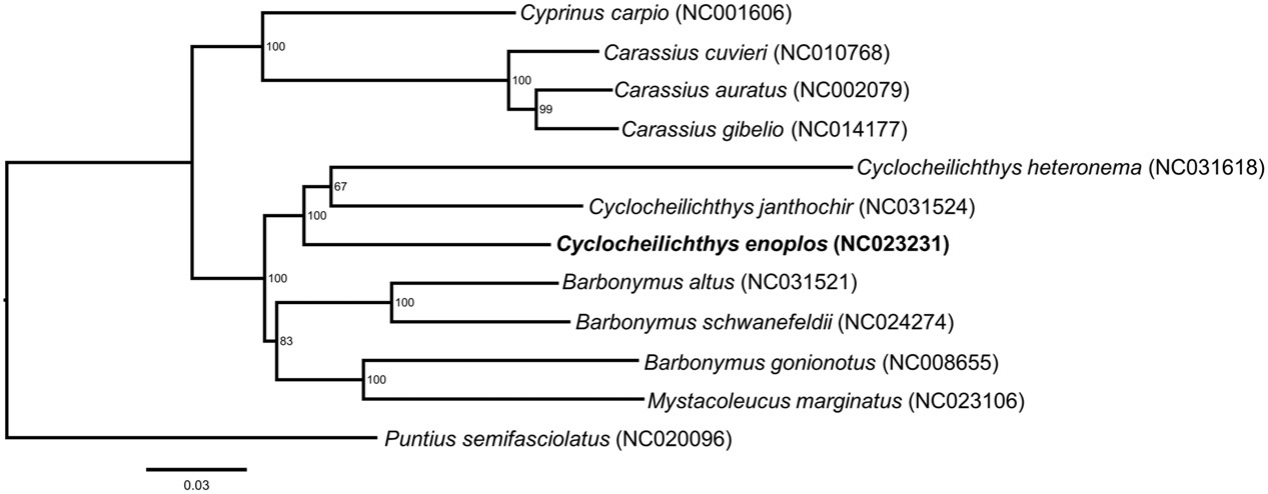
Maximum-likelihood (ML) phylogenetic tree of *C. enoplos* and the other 11 species using *P. semifasciolatus* as an outgroup. Number above each node indicates the ML bootstrap support values.
